# 2366 Urinary tract infections in children with kidney allografts: Risk factors and clinical consequences

**DOI:** 10.1017/cts.2018.145

**Published:** 2018-11-21

**Authors:** Annie Farrell, Larry Greenbaum, Traci Leong

**Affiliations:** 1 Emory University; 2 School of Medicine and Children’s Hospital of Atlanta, Emory University; 3 Rollins School of Public Health, Emory University

## Abstract

OBJECTIVES/SPECIFIC AIMS: Background: Renal transplantation (tx) is the optimal treatment for end-stage renal disease (ESRD) in children, but post-tx urinary tract infections (UTIs) may cause morbidity and reduce allograft survival. Objectives: To quantify the number and risk factors for UTIs in pediatric kidney tx recipients in preparation for an analysis of the morbidity and impact of UTIs on allograft survival. METHODS/STUDY POPULATION: Methods: We identified all patients who underwent kidney tx between 2001 and 2016 (n=390) at Children’s Healthcare of Atlanta (CHOA). Patients were included if they had >1 year of follow-up at CHOA. We conducted an IRB-approved, retrospective review of patient demographics, medical history, and tx outcomes in the 5 years following tx. RESULTS/ANTICIPATED RESULTS: Results: Of the 205 records reviewed to date, we identified 176 eligible patients (61.9% male). Mean age at tx was 11.7±5.5 years. In total, 58.5% had a deceased and 41.5% had a living kidney donor. Obstructive uropathy was the etiology of ESRD in 21.0%. Mean UTIs in all patients was 1.1/patient±2.7. On preliminary analysis, patients with a history of obstructive uropathy were more likely to develop a UTI than patients without (45.9% vs. 25.2%, *p*=0.014). There is a trend to more UTIs in patients with a history of obstructive uropathy compared with patients without (2.1±3.5 vs. 0.9±2.4, *p*=0.055). In males, there were more UTIs in patients with a history of obstructive uropathy compared to patients without (1.7±2.9 vs. 0.5±1.5, *p*=0.024). In all, 23.2% of all patients were on UTI prophylaxis post-tx; trimethoprim-sulfamethoxazole was the prophylactic antibiotic in 54.5%. DISCUSSION/SIGNIFICANCE OF IMPACT: Conclusions: UTIs are common post kidney tx in children, especially in those with a history of obstructive uropathy. The associated morbidity and impact on graft survival are unknown.

